# Ex-vivo porcine organs with a circulation pump are effective for teaching hemostatic skills

**DOI:** 10.1186/1749-7922-7-5

**Published:** 2012-03-09

**Authors:** Yoshimitsu Izawa, Shuji Hishikawa, Tomohiro Muronoi, Keisuke Yamashita, Masayuki Suzukawa, Alan T Lefor

**Affiliations:** 1 Department of Emergency Medicine, Jichi Medical University, Tochigi, Japan; 2 Department of Surgery, Jichi Medical University, Tochigi, Japan This manuscript was presented in part at the World Society of Emergency Surgery 1st World Congress on 2nd July 2010 in Bologna Italy

**Keywords:** Ex-vivo, Trauma surgery, Education, Porcine model

## Abstract

Surgical residents have insufficient opportunites to learn basic hemostatic skills from clinical experience alone. We designed an ex-vivo training system using porcine organs and a circulation pump to teach hemostatic skills. Residents were surveyed before and after the training and showed significant improvement in their self-confidence (1.83 ± 1.05 vs 3.33 ± 0.87, P < 0.01) on a 5 point Likert scale. This training may be effective to educate residents in basic hemostatic skills.

## Background

Simulation training for surgical skills has become essential around the world. Many methods including dry laboratories, simulators, cadavers, and live tissues have been used for basic surgical skill training, open surgery training, and laparoscopic training [1]. To improve trauma surgery education, many educational training courses have been developed. Specifically, many simulation courses such as Advanced Trauma Operative Management, Definitive Surgical Trauma Care, and Advanced Surgical Skills for Exposure in Trauma have been held around the world [2-7].

Among the various possible approaches, live animal training may be most suitable for teaching hemostatic skills [1]. However, these courses are expensive and it is difficult to provide repetitive training because they utilize live animal models necessitating general anesthesia, as well as much time and effort. Recently, the use of live animals is decreasing in surgical training. The validity of using a simulated model instead of live animals has been validated for chest tube placement and cricothyrotomy [8]. In addition, it is critically important to adopt the 3R approach to the use of animal models, including Reduction, Refinement and Replacement, originally described in 1959 [9].

Simulation training programs may not be suitable for certain kinds of training because the bleeding encountered is not similar to live animals. Ex-vivo training as a type of simulation for surgical education is a less realistic model of hemorrhage than a live animal. However, such courses may be relatively inexpensive and allow repetitive training [1].

Recently, with fewer opportunities to participate in live animal training due to economic and ethical aspects, and limited trauma operative experience during training, residents may not be able to learn adequate hemostatic skills in clinical trauma situations alone [10]. In order to improve the competency of residents in basic hemostatic skills in the trauma setting, we created this realistic, repetitive, and ethically-advantageous ex-vivo training model to teach hemostatic procedures using a circulation motor and ex-vivo porcine organs, providing an opportunity for residents to learn hemostatic skills.

## Materials and methods

This training was carried out in a humane manner after receiving approval from the Institutional Animal Experiment Committee of Jichi Medical University, and in accordance with the Institutional Regulation for Animal Experiments and Fundamental Guideline for Proper Conduct of Animal Experiment and Related Activities in Academic Research Institutions under the jurisdiction of the Ministry of Education, Culture, Sports, Science and Technology. Participants were recruited from among residents (PGY 2 through PGY 5) rotating in the Emergency Department at the time of the study. Participants were informed about the nature of the program and given the option to participate.

All animals used were specific pathogen free and were tested for the absence of Hepatitis E Virus. Animals were obtained from a breeder directly, and included Mexican and Chinese mini-pigs weighing 30-45 kg each, and treated in accordance with appropriate rules and regulations for the ethical care of laboratory animals. Previous experiments included various surgical procedures that would not introduce added risks to participants. Porcine hearts, kidneys, and inferior vena cavae (IVCs) were harvested from animals used in other experiments and stored cryogenically until the training sessions. On the day of the session, the frozen organs were thawed and connected to circulation pumps. Circulating water was mixed with red ink to simulate blood. All participants received didactic training with a one hour lecture, and were were surveyed regarding their confidence to perform the procedures before the laboratory session (Table 1).

**Table 1:** Self-Confidence Level of Participants Before and After Simulation Training

Time Measured	Mean	SD	P-Value
Pre-Course	1.83	1.05	< .01

Post-Course	3.33	0.87	

Self-Confidence measured on a 5-point Likert scale (1 = no confidence, 3 = Neither Confident nor lack of confidence, 5 = strong confidence)

**Figure 1:**
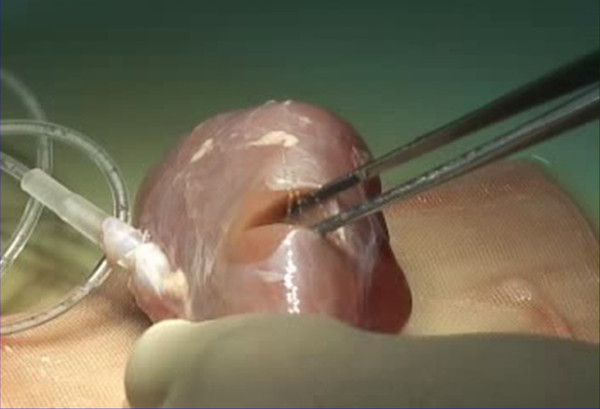
**A Renal cortex injury is made in a kidney connected to a circulation pump with saline circulating through the renal vessels**.

**Figure 2:**
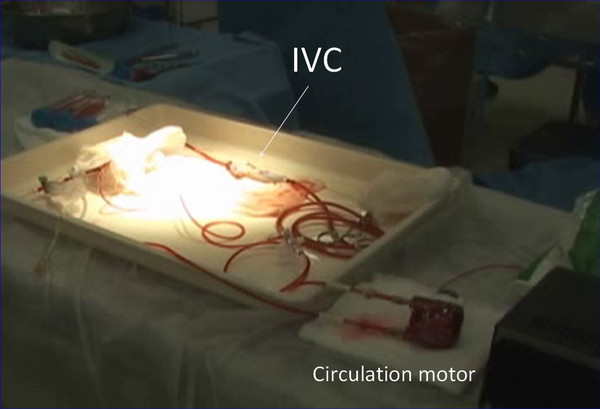
**An ex-vivo porcine inferior vena cava (IVC) is connected to a circulation pump for teaching hemostatic techniques**.

**Figure 3:**
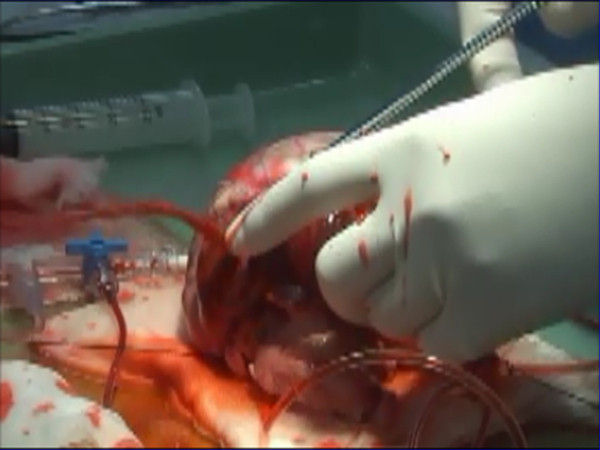
**An ex-vivo porcine heart is connected to a circulation pump for teaching hemostatic techniques**.

Participants then moved to the laboratory, and suture hemostasis was performed in the renal cortex (Figure 1), IVC (Figure 2) and the injured heart (Figure 3) while active bleeding was simulated by the flow of the circulating red water through native vessels. This was done under close supervision and mentorship by senior faculty in Emergency Surgery (YI, TM, KY, and SH). The dynamic nature of the bleeding simulation is easily seen in the Additional file 1: Vedio S1; Additional file 2: Vedio S2. Participants were given the opportunity to repeat the simulation, and to attempt different approaches to achieve hemostasis. The laboratory session lasted about 5 hours total, with each participant spending time with each of the three organs.

Following the training, participants were surveyed regarding their confidence and their opinion of the training. The survey used a 5-point Likert scale, with 1 indicating low confidence and 5 indicating the highest confidence. These results are shown in Tables 1 and 2.

**Table 2:** Participant Evaluation of the Course

Question	Mean Score	± SD
I understood the goals and objectives for this trauma ex-vivo training program	4.63	0.647

My interest in trauma care has increased	4.75	0.442

I am satisfied with this training	4.54	0.721

I would recommend this training to my colleagues	4.75	0.531

I would like to repeat this training	4.79	0.415

Repeating this training would make me more capable in torso trauma surgery	4.75	0.442

Scores shown on a 5-point Likert scale (1 = strongly disagree, 3 = neither agree nor disagree, 5 = strongly agree). *SD*, Standard Deviation

### Statistical Analysis

Survey data was analyzed by Wilcoxson rank-sign test (Excel, Microsoft Corp, Redmond WA USA), and is reported with mean, standard deviation, and p-value comparing the scores before and after training.

## Results

Twenty-four residents participated in this training program and performed hemostatic procedures. The training level of the residents included: PGY 2, 16 (67%), PGY 3, 6 (25.0%), PGY4, 1 (4%), and PGY5, 1 (4%). Their experience in trauma surgery as surgeon or assistant prior to this program included: no cases for 8 participants (33%), 1 ~ 5 cases for 13 participants (55%), 6 ~ 10 cases for 2 participants (8%), and 15 or more cases for 1 participant (4%).

Residents were divided into groups and the program for each group was conducted at a different time, to enable close faculty mentorship. In total, the sessions were conducted eight separate times. A questionnaire was given to all participants both before and after the program. Responses showed a significant (*p *< .01) improvement in self-confidence (Table 1) after the program compared to before the training. Each of the eight sessions utilized one porcine heart, one porcine vena cava and one or two porcine kidneys depending on the number of participants.

All participants were satisfied with the training they received, and gave very positive feedback concerning the program (Table 2).

## Discussion

In Japan, nearly all trauma patients are victims of blunt traumatic injuries, particularly from automobile accidents. There is essentially no penetrating trauma at all. The number of patients undergoing surgery for blunt injuries has decreased given improvements in automotive safety and design. Hemostatic procedures are one of the most important skills in trauma surgery. Surgical residents should master the crucial hemostatic skills to deal with the hemorrhage in trauma operations. However, they have few chances to learn hemostatic skills in actual clinical care, due to a paucity of operative cases as well as the hierarchical nature of training [10]. We sought to develop an effective simulation model to teach hemostatic skills to residents, and conducted ex-vivo training with a circulation pump to provide residents with a chance for basic hemostatic skill training.

Various types of simulation training exist in surgical education. Reznick et al described the features of the types of simulation available and concluded that live tissue is suitable for procedures requiring blood flow [1]. Live animal training may be ideal for for hemostatic skill training. Many trauma surgery courses held around the world utilize live tissue for learning hemostatic skills. However, these courses are generally expensive and do not allow repetitive experiences. Furthermore, from an ethical perspective, we must seek to reduce the use of live animals. The direct costs of this study were limited to the facility fee and the cost of consumable items such as sutures. The facility fee included the cost of storing the organs and use of instruments. There were no other associated direct costs.

Cadaver training, which demonstrates accurate anatomy, is suitable for learning complex surgical procedures [11] but cannot be used in realistic simulations for teaching hemostatic techniques because there is no bleeding. Though a virtual reality simulator is reusable and easy to prepare [12], its texture is far from realistic and its three-dimensional image is generally well simulated so that it is not a realistic model. Although some types of dry-models are useful for surgical training [13], they cannot make a realistic bleeding model.

The model used here maintains the texture of live tissue because actual organs are used. The freeze/thaw cycle did not change the tactile sensation of the tissue, nor did it destroy the large vessels with in the organs, notably the kidney in the model used here. Also, by utilizing a circulation pump, it provides a more realistic training situation than ex-vivo tissue alone, yet is much less expensive than live animal models. This study demonstrates the value of this hybrid model, which exists between using live animals and typical ex-vivo tissue models.

In this study, we selected the heart, kidney and vena cava for the models. Each organ was only used for one session, but by multiple participants. The organs were not re-frozen because the multiple repairs precluded their re-use. It may be possible to use other organs, such as spleen or liver. However, cannulation of the porcine splenic vessels may be difficult because of their size. The repair of the kidney affords a similar experience to that of a spleen or liver, but was preferred because of the increased number of organs as well as the size of the kidney being conducive to easy cannulation and handling compared to the liver.

Ex-vivo training with a circulation pump model is suitable for basic hemostatic practice for residents. This training is easy to prepare and allows residents to practice hemostatic skills repeatedly, which may lead to earlier mastery some skill. Furthermore, this training is clearly advantageous from the ethical point of view compared with live tissue training. The concept of 3R is crucial regarding the ethics of using animal tissue in medical research and education. This training contributed to the Replacement and Reduction components of the 3R principle. The design of this model satisfies both reality and ethics.

There are some limitations to the sense of reality encountered in this model. This training does not use blood so that coagulation is completely absent compared to live tissue. For example, during repair of the IVC injury in this model, the oozing from the needle holes cannot be stopped.

Another limitation is the lack of a physiologic effect of bleeding. For example, the cardiac injury repair is easier in this ex-vivo model than in a live animal because it cannot offer the same motion during systole as a live heart. Donias et al made a beating heart model in an ex-vivo setting for coronary artery anastomosis training using a foot pump [14]. The cardiac muscle does not contract by itself so that the reality of ex-vivo training is not the same as that in a live animal. Precise re-creation is impossible using this model, but the practice afforded here may facilitate learning with a live animal model and requires further study.

An important aspect of this training is the close faculty participation required. Each organ used constituted a "station" and we felt it was important to have each station manned by a faculty member throughout the training, such that the time faculty time requirement is significant. Including the lecture time (1 hour) and laboratory time (5 hours), a total of 16 person-hours of faculty time are needed to conduct the session.

The effectiveness of simulation training can be defined in several ways, such as improved clinical performance following simulation training, improved patient safety following such training, or effects on the practitioner. In a study of thoracostomy tube placement by medical students, Hishikawa and colleagues found that while there was no measurable effect on performance of the task in a live porcine model with or without prior simulation training, the students who underwent simulation training felt significantly more confident when performing the task on a live animal [15]. The improved confidence observed in the present study is felt to be a valid measure of effectiveness, as was shown in the thoracostomy tube study.

This ex-vivo training level is excellent for surgical residents. This model cannot re-create hemorrhage for complex hemostatic procedures such as hemorrhage of multiple origins, so experienced trauma surgeons may not be satisfied with this training. Further studies are needed to judge the effectiveness of this training at various levels of training.

## Conclusions

Ex-vivo tissue training with circulation pumps for teaching basic hemostatic skills in trauma was developed to increase residents' opportunities to learn these important skills, and serves as a hybrid model combining the realistic feel of tissue and the experience of bleeding without the need for live animals. This training improved the confidence of residents in hemostatic skills of trauma surgery, and is one of the ways to educate residents for basic hemostatic skills. The model employed is economical, effective, and respects the 3R principle of animal ethics. Continued evaluation of various teaching modalities is an important goal in surgical education. This study serves as the basis of future larger studies, which will investigate the objective benefits of simulation training for teaching hemostatic skills.

## Competing interests

The authors declare that they have no competing interests.

## Authors' contributions

YI: Conceived the trial, conducted the training, collected and analyzed data, prepared the manuscript, SH: Conducted the training, collected the data, prepared the manuscript, TM: conducted the training, collected and analyzed the data, KY: conducted the training, collected and analyzed the data, MS: conceived the trial, analyzed the data, prepared the manuscript, AL: conceived the trial, Analyzed the data, preparation of manuscript, All authors read and approved the final manuscript.

## Supplementary Material

Additional file 1 **Vedio S1**. Ex-vivo simulation of blood flow in a cardiac injury with a circulation pump. Click here for file

Additional file 2 **Vedio S2**. Ex-vivo simulation of blood flow in a renal injury with a circulation pump. Click here for file
